# Using Work Ability Index and work-related stress to evaluate the physical and mental fitness of Iranian telecom tower climbers

**DOI:** 10.5249/jivr.v10i2.996

**Published:** 2018-07

**Authors:** Ali Khavanin, Javad Malakouti, Vahid Gharibi, Narges Khanjani, Hamidreza Mokarami, Mohammad Hossein Ebrahimi

**Affiliations:** ^*a*^Department of Occupational Health Engineering, Faculty of Medical Sciences, Tarbiat Modares University, Tehran, Iran.; ^*b*^Faculty of Public Health, Qom University of Medical Sciences, Qom, Iran.; ^*c*^Environmental and Occupational Health Research Center, Shahroud University of Medical Sciences, Shahroud, Iran.; ^*d*^Environmental Health Engineering Research Center, Kerman University of Medical Sciences, Kerman, Iran.; ^*e*^Department of Ergonomics, Shiraz University of Medical Sciences, Shiraz, Iran.; ^*f*^Environmental and Occupational Health Research Center, Shahroud University of Medical Sciences, Shahroud, Iran.

**Keywords:** Work Ability Index, Work-related stress, Occupational health, Tower climbers

## Abstract

**Background:**

Working at height has always been considered as one of the most dangerous industrial activities. Workers' fitness including their physical and psychological ability plays a key role in prevention of occupational accidents. This research was conducted to investigate the physical and mental fitness of telecommunication tower climbers as well as their job stress.

**Methods:**

This cross-sectional study was conducted among employees of a contracting company which worked in the field of telecommunication tower installation in Iran during 2016. Sixty out of 93 workers voluntarily participated in this study. Data collection tools were the Persian version of the Work Ability Index (WAI), the Persian version of the Health and Safety Executive (HSE) Stress Indicator Tool and an author-developed measure to assess socio-demographic characteristics. Data was analyzed through statistical tests such as independent t-test, univariate analyses of variance (ANOVAs), Pearson’s correlation coefficient, and multivariate linear regression; using SPSS 19 software.

**Results:**

Workers' mean ± SD WAI score was 29.17± 10.33 and for work-related stress was 3.08 ± 1.80. There was a significant relation between WAI and educational level, job tenure, hours of sleep per day, regular exercise, and second job. The repression modeling explained 67.4% of the total variance (adjusted R2) of the WAI score. Among the dimensions of work related stress, control (β = 0.21) and changes (β = -0. 40) were significant predictors of the WAI score.

**Conclusions:**

To improve the worker’s work ability, intervention programs should focus on promoting level of job control, sleep quality and exercise. Additionally, implementing a comprehensive macroergonomics and participatory program for increase involvement the workers in organization changes should be considered.

## Introduction

Working at height has always been considered as one of the most dangerous industrial activities. According to the database of the Iranian Ministry of Labor, and Social Welfare, 3933 out of 10583 work-related accidents registered in 2012 were related to falls. These accidents led to the loss of 3257844 working days.^1^ Therefore it would be important to find the cause of these accidents and clarify effective factors on unsafe behaviors or acts, in order to conduct effective safety control measures.^2^ Despite the fact that there are not any official statistics of occupational accidents among Iranian telecommunication tower climbers, the Iranian Ministry of Energy reported that 130 tower climbers died and 900 were injured in the recent years.^3^

The concept of work ability index (WAI) is defined by Finnish Institute of Occupational Health (FIOH) as the worker's ability to work according to work requirements, health situation, and mental-thinking capabilities.^4^ The WAI reflects interactions between physical and mental abilities (individual characteristics), working conditions, employee performance capabilities, employees' health status and individual assessment of position in the organization and community (social characteristics).^5,6^ Occupational accidents were found to be the predictors of WAI. ^7^

The WAI is directly connected with workers' individual, physiological, mental and intellectual features and can significantly help employers to predict whether the applicant has the ability to work now and in the immediate future.^8,9^ The International Labor Organization (ILO) has reported the imposed work-related stress costs countries from 1 to 3.5 percent of GDP^10^ and introduced it as the better known phenomenon which threatens workers' health. 

According to the World Health Organization (WHO), more than a half of employees in industrial countries are suffering from work-related stress.^11^ Occupational Stress is considered as the second prevailing problem after pain.^12,13^ There is a significant negative relationship between work-related stress and job performance.^14^ Nowadays, workplaces are strong stimulants for creation of excitement and there are stressful situations which could cause problems such as dissatisfaction, employee absenteeism, decreased efficiency, and job turnover.^15^ Workers' suitability for work relates to their knowledge, skills, and physical and mental abilities that is significantly important for preventing occupational accidents. Considering these factors is necessary in occupational health and safety management standards.^16^

A literature search on studies conducted in Iran, showed that there was no study on work ability and job stress among Iranian tower climbers. However, it is essential to pay attention to this issue and the events which could happen to them. 

This study aims to investigate the relation between WAI and some socio-demographic variables and work-related stress, in order to evaluate the physical and mental fitness of Iranian telecom tower climbers.

## Methods 

This was a cross-sectional study. It is conducted among employees of a contracting company which worked in the field of telecommunication tower installation in Iran during 2016. Sixty out of 93 workers voluntarily participated in this study. The data collection tools included three questionnaires; including the Persian version of the WAI, the Persian version of the Health and Safety Executive (HSE) Stress Indicator Tool and an author-developed measure to assess socio-demographic characteristics.

**Work ability index (WAI) **

This index has 7 dimensions including: current work ability compared with the workers best lifetime working period, work ability according to the physical and mental nature of the job, number of current diagnosed diseases by a physician, personal thoughts about work incapability due to disease, sick leave during the past 12 months, personal prediction of work ability during the next two years and estimates about mental problems and harms related to disease.

The validity and reliability of the WAI questionnaire was determined in an Iranian study by Abdolalizadeh et al. and had a Cronbach's alpha coefficient equal to 0.79.^17^

The high reliability and validity of the WAI questionnaire and its application was approved in other studies.^18^ Based on the WAI questionnaire, the final score of an every worker varies from 7 to 49 and is calculated by the sum of scores for each question. Work ability index is classified into four groups which are weak (7-27), medium (28-36), good (37-43) and excellent (44-49).^19^

**Socio-demographic data**

The variables questioned in this study were job title, age (the total years of life from birth year), educational level (the last educational degree), marital status (person's state of being single, married, divorced, or widowed), sleep per day (the total hours of sleeping in a day), second job (second job condition) and job tenure (the total years of work). Exercise habit was divided into two classes; yes and no. Yes meant that they had a minimum habit of doing exercise, in which they sweat lightly for over 30 minutes each time, twice weekly, and for over a year.^20,21^ In order to calculate Body Mass Index (BMI) the weight of workers was measured with minimum clothing and no shoes using a digital scale and their height was measured using a measuring tape in straight standing position without shoes.

**HSE indicator tool **

This tool is used to survey work-related stress. It has been used in several studies.^5,22-24^ According to research by Cousins et al, the reliability coefficient of the HSE questionnaire is 0.7, and the Cronbach's alpha coefficient of subscales (dimensions) of this tool is from 0.63 to 0.83.^25^ The results of a study by Marzabadi and Gholami Fesharaki confirmed the validity and reliability of the Persian version of the HSE Stress indicator.^26^ There are 35 questions in this tool with 7 criteria including demand (8 items), control (6 items), managerial support (5 items), peer support (4 questions items), relationships (4 items), role (5 items) and change (3 items).

The items of the HSE questionnaire include a 5-point Likert Scale (never, rarely, sometimes, often and always). The score of each question range from 1 to 5, under which 1 indicates undesirable, and 5 refers to desirable state.

**Data gathering**

The subjects were first classified into 4-5 groups in order to collected data. Informed consent was obtained from all participants. Privacy and confidential issues were considered throughout the study. The study excluded those respondents who were not interested in being involved in the survey. Research objectives were explained for research groups before response to questionnaires, and then questionnaires were distributed among them. The study was approved by the ethics committee of Shahroud University of Medical Sciences, Iran (Ethics Code: 9524).

**Statistical analysis**

Statistical descriptive methods were used to indicate the characteristics of the research population.

Independent t-tests and univariate analyses of variance (ANOVAs) were used to assess the effects of socio-demographic variables on the WAI score. Finally, to predict the WAI score from the work-related stress dimensions of the HSE questionnaire, we used a linear multiple regression analysis. All analyses were done at the significance level of 0.05 by the help of SPSS 19 software (IBM Corp., Armonk, NY, USA).

## Results

A total of 60 of the 93 workers participated in the study. The number of tower climbers was 48 (80%) and the number of tower climbing supervisors was 12 (20%). The mean ± SD age, BMI, job tenure and sleep per day (hours) were 38.91±9.83 years, 24.92±2.49 Kg/m^2^, 8.63±7.44 years and 6.62±1.17 hours, respectively. 40 precent of participants had academic degrees, 33.3% had a high (secondary) school diploma, and 26.7% were elementary. Descriptive statistics of socio-demographic variables and their relation with the WAI are shown in Table 1 and 2.

**Table 1:** Means, SDs and correlation between the quantitative demographic variables and WAI among the studied subjects (n= 60).

Variables	Mean ± SD	r*	P-value
Age (years)	38.91 ± 9.83	0.458	<0.001
Job tenure (years)	8.63 ± 7.44	0.500	<0.001
Sleep per day (hours)	6.62 ± 1.17	0.514	<0.001
BMI (kg/m2)	24.9 ± 2.5	0.072	<0.001

* Pearson’s correlation coefficient

**Table 2:** Means, SDs and correlation between the qualitative demographic variables and WAI among the studied subjects (n= 60).

Variables		N (%)	Mean WAI ± SD	P-value
**Job title**				
	Worker	48 (80)	27.08 ±10.28	<0.001
	Supervisor	12(20)	37.54 ± 4.91	
**Educational Level**				
	Elementary	16 (26.7)	22.96 (7.77)	
	High school diploma	20 (33.3)	24.42 (9.09)	<0.001
	Associate degree	12 (20)	36.54 (8.77)	
	Bachelor and above	12 (20)	38.0 (5.29)	
**Marital status**				
	Single	8 (13.3)	31.12 (10.32)	0.691
	Married	49 (81.7)	29.56 (10.27)	
**Regular exercise**				
	Yes	22 (36.6)	35.65 (8.82)	0.023
	No	38 (63.3)	24.64 (9.23)	
**Second job**				
	Yes	27 (45)	22.39 (8.20)	<0.001
	No	33 (55)	34.42 (8.19)	

According to results of ANOVA and t-test, there is a significant relationship between educational level, regular exercises, and second job status with the mean WAI score. 

**Figure 1:**
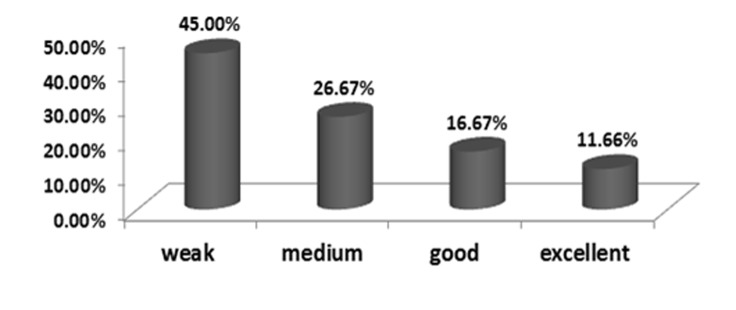
Distribution frequency of WAI groups.

Distribution frequency of classified WAI groups are shown in Figure 1.

The mean ± SD WAI score was 29.17 ± 10.33. It was 27.08 ± 10.28 in tower climbers, and 37.54 ± 4.91 in tower climbing supervisors. Also, the minimum and maximum scores were 13 and 47 in these groups.

According to another finding of this research, a second job is among factors which reduce the WAI among tower climbers so that 36.67% of employees with second jobs were classified into the weak group in terms of work ability. Statistical Pearson correlation coefficient (r) test was used to determine the effect of all seven indices on the total WAI score. According to results, the maximum direct effect belongs to the first dimension (current work ability compared with the best lifetime working period) (P <0.001, r=0.892) and the minimum direct effect belongs to the fifth dimension (sick leave during the past 12 months) (P <0.001, r=0.700). 

The mean final score of HSE work-related stress was 3.08±1.80. According to results of the Pearson correlation test, there was a significant correlation between work-related stress with WAI (P<0.001, r=0.686). 

The mean ± SD scores of the dimensions of HSE work related stress were as follows: demand (3.20 ± 1.27), control (2.91 ± 0.93), managerial support (2.92 ± 0.99), peer support (3.38 ± 1.74), relationships (2.80 ± 1.11), role (3.28 ± 1.15), and changes (3.10 ± 1.05). The results of linear regression are presented in Table 3.

**Table 3:** The results of multivariate linear regression analysis for work-related stress dimensions predicting the WAI score (n = 60).

Variables	B	SE	β	P-value
Control dimension	2.36	1.175	0.21	0.049
Changes dimension	3.94	1.04	0.40	<0.001
R2	0.707			
Adjusted R2	0.674			

* Adjusted results based on confounding variables including age, BMI, educational level, job tenure, second job status, hours of sleep per day, job title and regular exercise.

The results showed that after adjusting for demographic variables including age, BMI, educational level, job tenure, second job status, hours of sleep per day, job title and regular exercise; only two dimensions of work-related stress including control and changes were significant predictors of the WAI score. These variables explained 67% of the total variance (adjusted R2) of the WAI score.

## Discussion

The mean score of WAI for employees, who worked in the tower climbing industry were 29.17±10.33 which is in the lowest mean class. However, different studies reported WAI mean scores of 40.3±5.2 for medical staff of a teaching hospital in Tehran,^27^ 36.8 for bus drivers,^28^ 36.46±6.44 for fruit and vegetable booth workers,^29^ 38.37±5.80 for cement industry employees,^30^ 39.95± 6.1 for Iranian employees from different work sectors,^31^ 42.2±3.4 for physicians,^32^ 36.8 for bus driver, ^33^ and 40.9 for metal industry employees in the Netherlands, ^34^ and 40.7 for metal industry employees in Finland. ^35^ Furthermore, the mean WAI was 40.9 among construction workers in the Netherlands, 39 in police officers in Finland, and 40.6 in Belgian fire fighters. ^36^ Obviously, the mean scores of WAI were lower in tower climbers than the above-mentioned jobs. Low control and high physical demand over tasks, risky and time-consuming jobs can be the reasons for low mean WAI among various workers' communities.^37^ Difference in tower climbing supervisors' job nature with executive and operational affairs and also supervisors' higher educational levels were among the reasons for the difference between WAI in tower climbing supervisors (37.54) and tower climbers (27.08).

In this study, there was a significant relationship between age and the mean score of WAI that is consistent with the results of Guidi et al.^38^, Pouyakian et al. ^29^ and Mokarami et al., ^7^ but inconsistent with Hajizadeh et al.’s results. ^30^ Previous studies^39^ have found that the ability of individuals will diminish after age 40, which is consistent with the results of this study where the average age of people is under 40 and the work ability index is independent of age.

In this research, the significant direct relationship between educational level and WAI and a unit increase in educational level will lead to increase in WAI . For instance, 41.66% of workers with educational level of elementary and diploma were in the weak group, but only 3.33% of workers with academic degrees were in low levels of the index, and this indicates the role of educational in increasing work ability. This is consistent with research done by Eyvazlou et al.^40^, but is inconsistent with results from Pouyakian et al. ^29^ Academic education will probably increase individual skills for improving job quality and safety. 

The results indicated that the work ability increased with increasing job tenure that is in line with finding of Eyvazlou et al.^40^ However, it is inconsistent with finding of Pouyakian et al. ^29^ Mehrdad et al.^27^ and Sormunen et al.^41^ A suitable job should be based on individual knowledge, skill, experience, interest, characteristics and ability. Therefore it is essential to choose applicants correctly in order to achieve a high level of safety and productivity. 

Lifestyle-related factors including the hours of sleep per day and regular exercise, which are studied in this research, have a significant direct relationship with WAI. There is a positive correlation between sport and physical activity with WAI in studies by Sampaio et al.^28^, Kumashiro et al. ^34^, Nachiappan et al. ^42^ Regular exercise is an important part of a healthy lifestyle and helps to create and strengthen physical and mental health which improves individual work ability.

 We found a significant relationship between WAI and length of sleep, as one of the predictors of WAI, which is consistent with results reported by other researchers.^43,44^ Mokarami et al. described sleep quality is an important predictor for worker’s health and quality of life. ^45,46^

In this study, having a second job has a significant role in reducing work ability, so that among all studied groups and elements, the lowest mean score of WAI belonged to employees who had second jobs (WAI= 22.39). Lack of attention to physical conditions of the workplace, lack of physical safety, lack of total compensation of the current job and lack of job satisfaction are among the major factors pushing tower climbers to have second jobs.^47^ According to Iranian labor laws, every worker should work 8 hours per day. Having a second job can lead to low safety and job quality and can finally affect the health and work ability of workers.

From the above mentioned results, it can be concluded that WAI among tower climbers is significantly related to workers' life condition and quality; and the prominent parameters are length of sleep, exercise habits, second job status, work experience and educational level.

According to the results work-related stress and work ability index are correlated and increased occupational stress will reduce individual work ability, which is line with research done by Li et al.^48^, Karasek et al. ^49^, Martinez et al. ^50^ and Gharibi et al. ^5^

The results of regression modeling showed that “changes” was one of the negatively predicting factors of WAI. This variable is related to establishing changes in the workplace and workers ' knowledge about these changes. Misunderstanding of the quality/quantity of changes and their subsequent effects on the job and workplace can lead to stress in workers.^51^ The results of other studies indicate that if the worker is unprepared for changes (work environment and work resources) and he is not given enough knowledge before applying these changes, such changes can cause occupational stress.^52-54^ In accordance with Hackman and Oldham’s job characteristics model, feedback and autonomy are two main dimensions that should be considered in every job design. Without these dimensions, psychological problems such as decreasing job satisfaction, increasing work absence and job turnover might be observed. ^55^ Therefore, organizational managers need to inform their workers about changes and the results/effects of these changes especially those which have a direct impact on their jobs.

According to this research, the subscale of control was another work-related stress predictor for WAI; and this is in line with the results obtained by other researchers.^38^ Lack of control over job activities such as ability to rest at a desired time, work pace, and self-time management had a negative impact on the WAI. ^9,38^ According to Karasek et al., jobs with high demand and low control are highly stressful jobs and cause stress and disease.^49^ Lee and Wang investigated job stress and its relevant factors in nurses and reported that nurses' workload and higher responsibility were the main sources of their job stress.^55^

The small population and using self-reporting tools were the main limitations of this study. This research is the first study in the field of health and safety of tower climbers in Iran. It is necessary to carry out more extensive research studies in order to improve their health and safety.

## Conclusions

This study demonstrated associations between WAI and lack of exercise, poor sleep quality, poor job control and poor involvement in organization changes. So, intervention programs should focus on promoting level of job control, sleep quality and exercise. Additionally, implementing a comprehensive macroergonomics and participatory program for increase involvement the workers in organization changes should be considered. 

**Acknowledgments**

This study was supported by Grant No. 9524 from Shahroud University of Medical Sciences. The authors appreciate Shahroud University of Medical Sciences for funding and supporting this project.

## References

[1] Alizadeh SS (2015). Estimation of economic costs of accidents at work in Iran: a case study of occupational accidents in 2012. Int J Occup Environ Med.

[2] Khosravi Y, Asilian-Mahabadi H, Hassanzadeh-Rangi N, Hajizadeh E, Gharibi V (2015). Why construction workers involve in unsafe behavior? Development and cross-validation of a structural model. Iran Occupational Health Journal.

[3] Iran’s ministry of energy. Information Center of the Ministry of Energy: Death tower climber in Iran, 2015, http://news.moe.gov.ir/Detail.aspx?anwid=18562, accessed 7 July 2018.

[4] Ilmarinen J, Rantanen J (1999). Promotion of work ability during ageing. American Journal of Industrial Medicine.

[5] Gharibi V, Mokarami H, Taban A, Aval MY, Samimi K, Salesi M (2016). Effects of work-related stress on work ability index among Iranian workers. Saf Health Work.

[6] Gould R, Ilmarinen J, Järvisalo J, Koskinen S. Dimension of work ability: Results of the Health 2000 Survey. 2008, Finnish Centre of Pensions, The Social Insurance Institution, National Public Health Institute, Finnish Institute of Occupational Health, Helsinki.

[7] Mokarami H, Mortazavi SB, Asgari A, Choobineh A, Stallones L (2017). Multiple dimensions of work-related risk factors and their relationship to work ability among industrial workers in Iran. International Journal of Occupational Safety and Ergonomics.

[8] Alexopoulos EC, Merekoulias G, Gnardellis C, Jelastopulu E (2013). Work ability index: validation of the Greek version and descriptive data in heavy industry employees. British Journal of Medicine and Medical Research.

[9] van den Berg TIJ, Alavinia SM, Bredt FJ, Lindeboom D, Elders LA, Burdorf A (2008). The influence of psychosocial factors at work and life style on health and work ability among professional workers. Int Arch Occup Environ Health.

[10] Zare M, Abedi K, Halvani GH, Barkhordari A, Aminipour M (2009). Prevalence of job stress among staff of the ports and sailing corporation of Hormozgan and its relation to non-fatal accidents. Journal of Shahid Sadoughi University of Medical Sciences.

[11] Torshizi L, Ahmadi F (2011). Job Stressors From Clinical Nurses’perspective. Iran Journal Of Nursing.

[12] Antigoni F, Pediaditaki O, Dimitrios T (2011). Nursing staff under heavy stress: focus on Greece A critical review. International Journal of Caring Sciences.

[13] Milutinović D1, Golubović B, Brkić N, Prokeš B (2012). Professional stress and health among critical care nurses in Serbia. Arh Hig Rada Toksikol.

[14] Vosoughi-niri A, Rohollahi A, Mohamad HH (2016). A survey of effect of job stress on general health and job performance on Air Traffic Controllers (ATC).. Iran Occupational Health Journal.

[15] Samadi H, Samadi H (2015). An investigation of relation between organizational justice and its components with employs job stress level in the workplace organization. Iran Occupational Health.

[16] Mokarami H, Choobineh A, Nazifi M (2017). A systematic review on the available questionnaires for the assessment of work-related stressors. Iran Occupational Health Journal.

[17] Abdolalizadeh M, Arastoo AA, Ghsemzadeh R, Montazeri A, Ahmadi K, Azizi A (2012). The psychometric properties of an Iranian translation of the Work Ability Index (WAI) questionnaire. J Occup Rehabil.

[18] Silva Junior SH, Vasconcelos AG, Griep RH, Rotenberg L (2013). [Test-retest reliability of the Work Ability Index (WAI) in nursing workers]. Rev Bras Epidemiol.

[19] Ilmarinen J (2007). The Work Ability Index (WAI).. Occupational Medicine.

[20] Minami H, Furukawa S, Sakai T, Niiya T, Miyaoka H, Miyake T (2018). Physical activity and prevalence of erectile dysfunction in Japanese patients with type 2 diabetes mellitus: The Dogo Study. J Diabetes Investig.

[21] Kawakami R, Miyachi M (2010). [Validity of a standard questionnaire to assess physical activity for specific medical checkups and health guidance]. Nihon Koshu Eisei Zasshi.

[22] Toderi S, Balducci C (2015). HSE management standards indicator tool and positive work-related outcomes. International Journal of Workplace Health Management.

[23] Nasiry Zarrin Ghabaee D, Haresabadi M, Bagheri Nesami M, Esmaeili R, Talebpour Amiri F (2016). Musculoskeletal disorders in nurses and their relationship with occupation-related stress. J Mazandaran Univ Med Sci.

[24] Gharibi V, Malakouti J, Arsang JS, Gholami A (2013). Prevalence of occupational stress and its relationship to individual characteristics in tunneling industry workers. Health System Research.

[25] Cousins R, MacKay CJ, Clarke SD, Kelly PJ, McCaig RH (2004). ‘Management Standards’ and work-related stress in the UK: Practical development. Work & Stress.

[26] Azad ME, Gholami FM (2011). Reliability and validity assessment for the HSE job stress questionnaire. Journal of Behavioral Sciences.

[27] Mehrdad R, Mazloumi A, Arshi S, Kazemi Z (2016). Work ability index among healthcare personnel in a university hospital in Tehran, Iran. Work.

[28] Sampaio RF, Coelho CM, Barbosa FB, Mancini MC, Parreira VF (2009). Work ability and stress in a bus transportation company in Belo Horizonte, Brazil. Ciência & Saúde Coletiva.

[29] Poyakian M, Zakerian SA, Avakh A, Mohamadian F, Kangavari M (2015). Worker’s work ability index in the fruit and vegetable stands in Tehran in 2014. Pajouhan Scientific Journal.

[30] Hajizadeh F, Motamedzade M, Golmohammadi R, Soltanian A (2015). Work ability assessment and its relationship with severity of musculoskeletal disorders among workers in a cement plant. Journal of Occupational Hygiene Engineering.

[31] Koohpayezadeh J, Kabir Mokamelkhah E, Alavinia MA, KarimiFarshi L, Akbari F (2014). Study on predictive value of work ability index to predict sick leave and disability caused by Work. Razi Journal of Medical Sciences.

[32] Camerino D, Conway PM, van der Heijden B, van der Schoot E, Pokorski J, Estryn-Behar M (2005). The role of job alienation in work ability deterioration and unhealthy ageing. San Diego: Elsevier.

[33] Kloimüller I, Karazman R, Geissler H, Karazman-Morawetz I, Haupt H (2000). The relation of age, work ability index and stress-inducing factors among bus drivers. International Journal of Industrial Ergonomics.

[34] Kumashiro M. Promotion of work ability towards productive agin. Selected Papers of the 3rd International Symposium on Work Ability; 22-24 October 2007, Hanoi, Vietnam. CRC Press, 2008.

[35] Tuomi K, Vanhala S, Nykyri E, Janhonen M (2004). Organizational practices, work demands and the well-being of employees: a follow-up study in the metal industry and retail trade. Occup Med (Lond).

[36] Alavinia SM, de Boer AG, van Duivenbooden JC, Frings-Dresen MH, Burdorf A (2009). Determinants of work ability and its predictive value for disability. Occup Med (Lond).

[37] van den Berg TI, Elders LA, de Zwart BC, Burdorf A (2009). The effects of work-related and individual factors on the Work Ability Index: a systematic review. Occup Environ Med.

[38] Guidi S, Bagnara S, Fichera G (2012). The HSE indicator tool, psychological distress and work ability. Occupational Medicine.

[39] Sluiter JK (2006). High-demand jobs: age-related diversity in work ability?. Appl Ergon.

[40] Eyvazlou M, Mazloumi A, Farshad A, Hoseini F (2012). Analytical evaluation of work ability index and its determining factors among workers of a car manufacturing industry. Iran Occupational Health.

[41] Sormunen E1, Remes J, Hassi J, Pienimäki T, Rintamäki H (2009). Factors associated with self-estimated work ability and musculoskeletal symptoms among male and female workers in cooled food-processing facilities. Ind Health.

[42] Nachiappan N, Harrison J (2005). Work ability among health care workers in the United Kingdom: A pilot. International Congress Series.

[43] Lian Y, Xiao J, Liu Y, Ning L, Guan S, Ge H (2015). Associations between insomnia, sleep duration and poor work ability. Journal of Psychosomatic Research.

[44] Lindholm H. Physiological determinants and assessment of stress and recovery among media workers. Helsinki, Finland: Finnish Institute of Occupational Health. 2013.

[45] Mokarami HR, Taghavi SM, Taban E (2016). Psychosocial factors and their relationship to health-related quality of life in an industrial factory in Yasuj City. Iran Occupational Health.

[46] Mokarami H, Stallones L, Nazifi M, Taghavi SM (2016). The role of psychosocial and physical work-related factors on the health-related quality of life of Iranian industrial workers. Work.

[47] Salari H, Ahmadi Y, Paktinat E (2013). Assessing factors influenced on teaching hospital staff tendency to incentives' in second job. Quarterly Journal of Nursing Management.

[48] Li H, Liu Z, Liu R, Li L, Lin A (2016). The relationship between work stress and work ability among power supply workers in Guangdong, China: a cross-sectional study. BMC Public Health.

[49] Karasek R, Brisson C, Kawakami N, Houtman I, Bongers P, Amick B (1998). The Job Content Questionnaire (JCQ): an instrument for internationally comparative assessments of psychosocial job characteristics. J Occup Health Psychol.

[50] Carmen Martinez M, da Silva Alexandre T, Dias De Oliveira Latorre MDR, Marina Fischer F (2016). Longitudinal associations between stressors and work ability in hospital workers. Chronobiol Int.

[51] Edwards J, Webster S, Van Laar D, Easton S (2008). Psychometric analysis of the UK Health and Safety Executive's Management Standards work-related stress Indicator Tool. Work & Stress.

[52] MacKay CJ, Cousins R, Kelly PJ, Lee S, McCAIG RH (2004). ‘Management Standards’ and work-related stress in the UK: policy background and science. Work & Stress.

[53] Hackett A, Palmer S, Farrants J (2009). Phase 1 of an investigation into the levels of stress in United Kingdom hospice services. Int J Palliat Nurs.

[54] Hackman JR, Oldham GR (1975). Development of the job diagnostic survey. Journal of Applied Psychology.

[55] Lee I, Wang HH (2002). Perceived occupational stress and related factors in public health nurses. J Nurs Res.

